# Role of Home-based primary care in perceived care sufficiency and depressive symptoms in older adults

**DOI:** 10.1093/geroni/igaf122.2443

**Published:** 2025-12-31

**Authors:** Jihee Choi, Bokyoung Choi, Jihwan Lee, Chang-O Kim, Soong-nang Jang

**Affiliations:** Institute for Community Care and Health Equity, Seoul, Republic of Korea; Chung-Ang University, Seoul, Republic of Korea; Chung-Ang University, Seoul, Republic of Korea; Institute for Community Care and Health Equity, Seoul, Republic of Korea; Chung-Ang University, Seoul, Republic of Korea

## Abstract

Background With the global increase in the aging population, the importance of community-based care and Aging in Place (AIP) has been emphasized. The study aimed to investigate the role of home-based primary care in the association between perceived care sufficiency and depressive symptoms among older adults in South Korea. Methods We conducted a survey of the ‘Home-Based Primary Care Cohort (HBPC). One survey has been completed by March 2024, the second survey by September 2024 and the third survey by March 2025. Study population was community-dwelling older adults aged 65 years and older who were receiving long-term care services. Perceived care sufficiency was assessed based on participant’s self-reported. Depressive symptoms were measured using GDS. Additionally, this study examined how the frequency of HBPC services moderated the relationship between subjective care sufficiency and depressive symptoms. Multiple linear regression models were applied to calculate. Results Among older adults receiving HBPC services, 61.4% (N = 202) reported that their caregiving hours were insufficient, and 82% (N = 270) exhibited depressive symptoms. A significant association was found between perceived care sufficiency and depressive symptoms (β = 0.89, p = 0.011). However, as the frequency of HBPC service utilization increased, the relationship between perceived care sufficiency and depressive symptoms became nonsignificant. Conclusions These findings emphasize the importance of ensuring perceived care sufficiency within the community to reduce depression among older adults. HBPC can play a role in designing future community-based care systems. Furthermore, HBPC may serve as a key resource in establishing an integrated community care system.

